# Supplementary material for: The dynamics of men's cooperation and social status in a small-scale society
Source: Proc Biol Sci. 2019 Aug 7;286(1908):20191367. doi: 10.1098/rspb.2019.1367 (PMC6710581; doi:10.1098/rspb.2019.1367)
Supplement: Supplementary Material for “The dynamics of men's cooperation and social status in a small-scale society” [file rspb20191367supp1.docx]

**Supplementary Material for “The dynamics of men’s cooperation and social status in a small-scale society”**

**Proceedings of the Royal Society B**

**DOI: 10.1098/rspb.2019.1367**

Christopher R. von Rueden^a*†^, Daniel Redhead^b,c*†^, Rick O’Gorman^c^, Hillard Kaplan^d^, Michael Gurven^e^

^a^Jepson School of Leadership Studies, University of Richmond, 221 Richmond Way, Richmond, VA 23173, USA. E-mail: [cvonrued@richmond.edu](mailto:cvonrued@richmond.edu).

^b^Department of Human Behavior, Ecology and Culture, Max Planck Institute for Evolutionary Anthropology, Leipzig, Germany. E-mail: [daniel_redhead@eva.mpg.de](mailto:daniel_redhead@eva.mpg.de)

^c^Department of Psychology, University of Essex, Wivenhoe Park, Colchester CO4 3SQ, UK

^d^Economic Science Institute, Chapman University, One University Drive, Orange, CA 92866 USA

^e^Department of Anthropology, University of California, Santa Barbara, CA 93106, USA

^*^To whom correspondence should be addressed

^†^Joint first author

***Photo Ranking of Status***

Each ranker evaluated the photos with no one else present but CvR and a non-Tsimane research assistant. The rankers were made aware of the confidentiality of their individual rankings. Verbal instructions were given by CvR in the Tsimane language. The instructions were translated from Spanish and then, as a test of the accuracy of translation, back-translated into Spanish by Tsimane’ men from other communities.

The particular photos evaluated by each ranker were selected according to a matrix based on a projective plane. We used a matrix whose number of unique values best corresponded to the number of adult men (21 years of age or older) to be ranked. Photos of men in the community aged 19 or 20 were added to make up the difference. A matrix based on the projective plane of order 8 (containing 73 unique numbers) was used in 2009 and in village 2. A matrix based on the projective plane of order 9 (containing 91 unique numbers) was used in 2014 and 2017.

We ensured that no ranker received a photo array in which they had to rank themselves. For every array presented, the photos were shuffled, and the order of the photos after shuffling determined the order in which they were placed in front of the ranker. Rankers were then asked to rearrange the photos into a line however they saw fit, with the highest ranked photo on the right and to its left lower ranking photos in succession.

***Factor Analysis***

Our analysis of status, income, and the size/strength measures as distinct attributes of actors is supported by a maximum likelihood factor analysis with promax (oblique) rotation. Included in the analysis were the two photo-ranked status measures, income, height, weight, and upper body strength from the first wave of data collection in village 1. The factor analysis was suitable for the items presented, with a Kaiser-Meyer-Olkin’s measure of sampling adequacy of 0.60 and a significant Bartlett’s test of sphericity ($\chi^{2}\left( 15 \right)=109.21, p<0.001).$Furthermore, the communalities between most factors were above the recommended value of 0.3, whilst upper body strength was 0.293 and income 0.169. All diagonals within the anti-image correlation matrix were above the recommended value of 0.5.

We extracted two correlated latent factors. As shown in Table S1, influence and respect loaded highly and positively onto the first factor, while the size and strength measures loaded positively onto the second factor. Although income loaded onto the status factor, it did not meet a minimum loading of 0.40 and was thus retained as a covariate apart from status and the size/strength measures. The status and size/strength factors were correlated (*r* = 0.37) and had eigenvalues of 2.75 and 1.23, respectively. The extracted factors accounted for 67% of the total variance (46% by status and 21% physical size and strength).

***Network Descriptives***

After censoring for individuals present in only one time wave, the composition of the cooperation network in village 1 initially comprised 60 actors, increases to 74 actors in time wave two and falls to 70 actors in the final time wave. The average number of cooperation partner nominations ranged between 3.4 and 7.8 and network density at each time point was 0.087 (wave 1), 0.044 (wave 2) and 0.072 (wave 3). The Jaccard index—a measure of tie stability between time waves—indicated that the network was relatively stable despite the significant time that elapsed between waves. 20.5% of ties remained the same between time wave 1 and 2, and 30.1% remained the same between time wave 2 and 3.

***Covariate Descriptives***

To fit the current data requirements for SAOMs [1], status and income were transformed into ordinal percentile rankings with 10 levels that were approximately equally distributed. This transformation ensured that 1 encompassed the lowest scorers (the 10^th^ percentile) and 10 the highest scorers (the 90^th^ percentile). Kinship was treated as an undirected network, where binary edges within the network indicated whether the respective dyad were close kin (indicated by 1) or not (indicated by 0). See Table S2 for descriptive statistics for all covariates included in the SAOM.

***Stochastic Actor-Oriented Model (SAOM) Description and Specification***

SAOMs estimate latent temporal changes in networks and actor behaviors, as outcomes of a sequence of decisions made by the actors that comprise the networks. For further elaboration and mathematical description see [1-5]. To do this, SAOMs simulate and estimate changes in the initial observed data that result in the final observed data. Change is assumed to occur in continuous time through a Markov process, and, unlike other longitudinal network models (i.e. Temporal Exponential Random Graph Models [6]), actors are given agency and are assumed to control their outgoing ties through a succession of steps. The opportunities for actors to make such changes are decomposed into the smallest possible step, termed a microstep, and are measured by a rate function [3]. During a microstep, an actor is randomly selected and has the opportunity to create, terminate or maintain a tie within the network, or increase, decrease or maintain a dependent behavior. When presented this opportunity for change, an actor evaluates all of their possible choices given the composition of the current network. Choices are weighted based on endogenous structural processes (e.g. reciprocity and transitivity) and exogenous mechanisms (e.g. to cooperate with kin). The factors that may be driving the network change and behavior change are measured using evaluation (or objective) functions, which are defined as the linear functions below (Equations 1 and 2).

$$1) f_{i}^{x}(\beta, x, z)= \sum_{k} \beta s_{ki}^{x}(x, z)$$

$$2) f_{i}^{z}(\beta, x, z)= \sum_{k} \beta_{k}^{z}s_{ki}^{z}(x, z)$$

For every actor, $i$, the values of the objective functions for network change and for change in a dependent behavior are represented by $f_{i}^{x}(\beta, x, z)$ and $f_{i}^{z}\left( \beta, x, z \right)$, respectively. These values depend on the state of the network $x$ and the actor’s behavior $z$. $\beta_{k}^{x}$ and $\beta_{k}^{z}$weights are the statistical parameters and the functions $s_{ki}^{x}$and $s_{ki}^{z}$are a combination of effects that may explain the change in network or behavior (see Table S3 for a description of the effects included within our full model). Actors will make a choice that is most likely to produce the highest values of their objective functions $f_{i}^{x}$ and $f_{i}^{z}$, with a small amount of randomness [7]. Positive $\beta_{k}^{x}$ and $\beta_{k}^{z}$ estimates indicate that a parameter makes a positive contribution to the objective function and therefore increases the average tendency for actors to create and maintain ties. Negative parameter estimates suggest the converse and estimates equal to 0 indicate that the effect may have no substantial impact.

Method of moments is used for parameter estimation, which simulates the change process –conditioned on the first observation—and models the network and behavior statistics to fit the values of the measured network and behavior. See [5] for a detailed overview of statistical specification and estimation procedure. Convergence of the model indicates that the parameter estimates are able to very closely simulate the actual measured networks and behaviors.

*Within-Network Effects*. Five effects were included to capture and control for the structural tendencies of the cooperation network dynamics (see Table S3). The first, outdegree, measured how selective individuals were in choosing cooperation partners. The tendency to reciprocate cooperation partnerships and to form transitive groups (i.e. if $i$ *→* $j$ and $j$ *→* $h$, then $i$ *→* $h$) were also included. Two-degree related effects, indegree popularity and outdegree activity, were included to capture the tendency to differentiate in degree, which assessed whether an actor’s anterior indegree/outdegree predicted their future indegree/outdegree.

*Covariate Effects.* A kinship network (combining affinal and consanguineal kin) was modeled as a binary dyadic covariate. An outdegree (ego), indegree (alter), and similarity effect were included to test our hypotheses regarding the impact that status has on cooperation dynamics. The status outdegree effect measures the impact of an actor’s status on the creation and maintenance of outgoing cooperation ties, i.e. the actor’s nomination of other men who cooperated with that actor. The status indegree effect measures the impact of an actor’s status on the creation and maintenance of incoming cooperation partner ties, i.e. nominations of the actor as a cooperator by other men. The similarity effect captures the selection of cooperation partners based on similarity in status**.** Outdegree, indegree, and similarity effects were also included in the model to assess how physical strength and size, income, and log age affected choice in cooperation partners.

*Status Dynamics*. We included the status average alter effect to assess our hypothesis about the effects of the cooperation network dynamics on status change. The status average alter effect measures how the status of individuals nominating an actor as a cooperation partner influences the actor’s status. Several effects were also modelled to fully partial the influence of cooperation on status. The first two, linear and quadratic shape effects, capture the linear trajectory of status and the feedback effect of status on itself, respectively. Effects on status change from physical strength and size, income, and age were also estimated.

***Robustness Checks***

To assess the sensitivity of our network influence finding to different formulations of network influence, we specified three additional models (see Table S4-S6 for estimates from these models). We estimated a model that included a total alter effect (i.e. the sum of the status’ of individuals that a focal actor has ties with). As shown in Table S4, the total alter effect was not substantially different from the average alter effect. To ensure that both the average and total alter effects are not solely being driven by network influence from incoming ties, we estimated two further models where we respecified the respective effects to measure the average in-alter and total in-alter (i.e. the sum or average of the status’ of alters that nominate the focal actor as a cooperation partner). Again, as shown in Tables S5 and S6, the estimates are qualitatively similar to the average alter effect.

To further examine the impact that general indegree and outdegree have on status dynamics (e.g. how an actor’s position in the network was associated with increased or decreased status), we sought to specify a model that included effects of differentiation in incoming or outgoing ties across actors on changes (or lack thereof) in status. However, we encountered difficulties in estimation when we included these indegree and outdegree effects. To bypass these issues, we specified a model where the indegree and outdegree parameters were fixed at 0, and used a score-type test that assessed whether the parameters make a substantial contribution to the objective function (See [1] for more information). Results of these score-type tests suggested that indegree may have a marginal association with increases in status, while outdegree seemed to have no real association with changes in status.

***Exponential random graph model (ERGM) description and results***

To assess the reliability of the SAOM results, an exponential random graph model (ERGM) was conducted on comparable cross-sectional data in a second Tsimane village. See Figure S1 for a depiction of the cooperation network in this village. ERGMs assess the probability of tie formation in a manner similar to generalized logistic models and allow for the inclusion of endogenous (structural) and exogenous (covariate) parameters that impact the network at an individual (actor/node) level, the dyadic (partner) level and network (group) level. For detailed outlines of ERGMs see [8-10]. As ERGMs cannot handle missing covariate data, we imputed missing data for status (18% missing), physical strength and size (7% missing), and income (1% missing) using a Bayesian Gaussian copula approach in R package sbgcop v.0.980 [11,12]. The missing status data in this community is due to the absence of several men, who only reside in the community sporadically, when residents were photographed for the status rankings. To assess the impact that imputation had on our ERGM results, we removed cases that had missing covariate data and estimated the same model on the subset of the data that had no missing data. Results were qualitatively similar (See Table S9 for results of this model).

The ERGM was conducted in the statnet suite of packages v.2018.10 [13] in R v.3.5.2 [14]. The model was built using available guidelines [15,16]. The model includes an edges parameter, which is analogous to the outdegree of our SAOM, a reciprocity parameter and a parameter assessing the tendency to form transitive groups (GWESP: which had a decay of 0.1 and was not fixed). We included node covariate effects to assess the impact that status, income, physical strength and size, and age had on cooperation tie formation. Directed terms (node indegree/node outdegree) were specified as the cooperation network was directed. An edge covariate effect was specified to test the impact that close kinship had on cooperation ties. See Table S7 for covariate descriptives. The final model reported is the most comparable to our SAOM and has the best model fit. The model had an MCMC sample size of 40,000 with a burn-in of 20,000 and an interval of 800. There was no sign of model degeneracy and there was adequate goodness-of-fit.

Results from the ERGM provide cross-sectional support for our SAOM (see Table S8) in a second village. The structural processes have a comparably large impact on cooperation ties relative to our SAOM, with a negative edges parameter $(OR =0.02, CI = [0.01, 0.05])$, reciprocity substantially increasing the odds of sending a tie $(OR =4.09, CI = [2.80, 5.97])$ and transitive groups tending to form $(OR =2.82, CI = [2.28, 3.49])$. There were increased odds for cooperation partnerships forming between close kin $(OR =5.83, CI = [4.86, 7.00])$. Status increased the odds of being nominated as a cooperation partner $(OR =1.05, CI = [1.01, 1.09])$, but decreased the odds of nominating others as cooperation partners $(OR =0.95, CI = [0.92, 0.99])$. A similar relationship was found between physical strength and size and cooperation. Physical strength and size increased the odds of receiving ties $(OR =1.28, CI = [1.12, 1.47])$ but marginally decreased the odds of sending ties $(OR =0.88, CI = [0.77, 1.01])$. Neither income nor log age had a substantial impact on indegree or outdegree nominations in this village.

**Figure S1**

The cooperation network in village 2 (*n* = 89; Avg. Degree= 12.18; Density= 0.069). Node size indicates the number of indegree nominations an individual received for sharing food or assisting in hunting, fishing, logging, or horticultural labor. Node color indicates the individual’s status, such that darker colors reflect higher status. Arrows indicate the directionality of ties, i.e. incoming arrows indicate receipt of cooperation nominations.

**
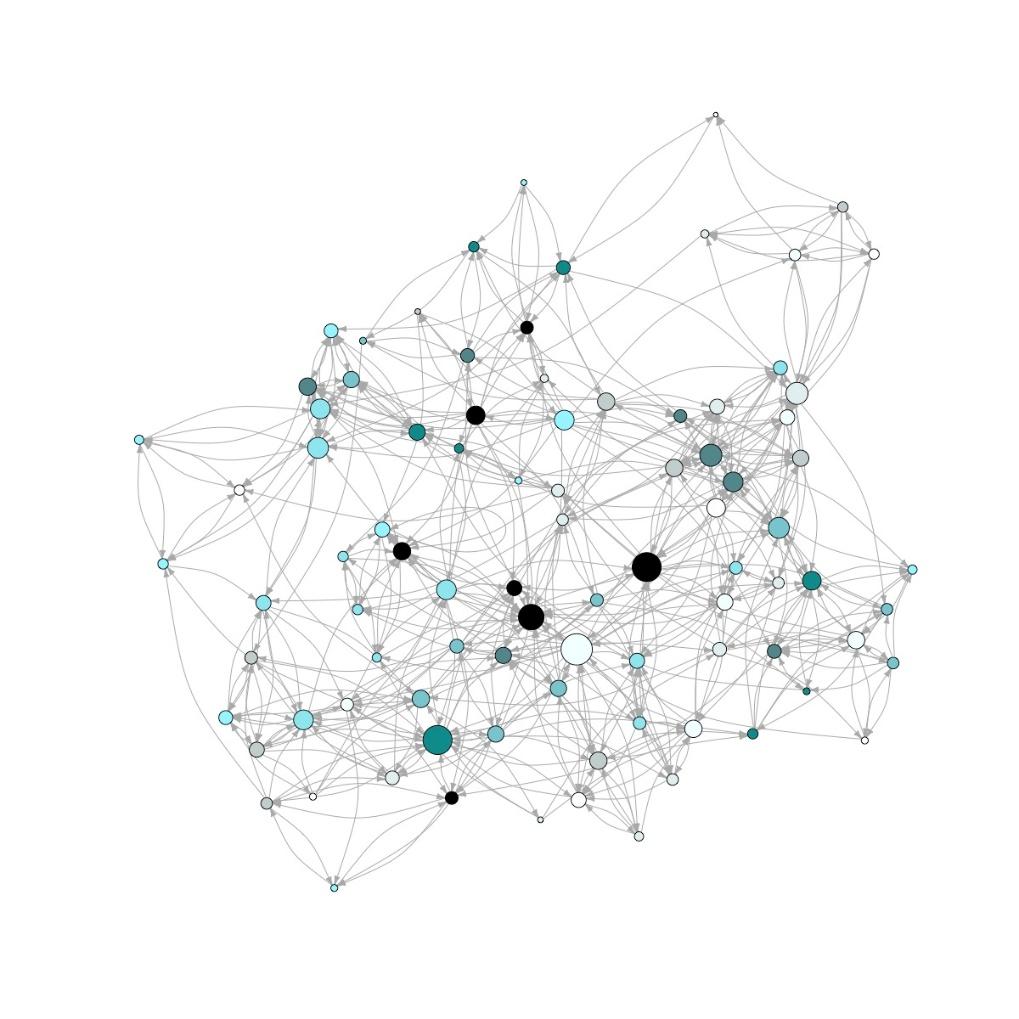
**

**Table S1**

Structure matrix from factor analysis of status and actor covariates, from first wave of data collection in village 1.

|  | | |
| --- | --- | --- |
| Item | Factor | |
|  | 1 | 2 |
| Influence | .999 | .349 |
| Respect | .803 | .450 |
| Income | .398 | .112 |
| Height | .219 | .987 |
| Weight | .426 | .629 |
| Upper body strength | .330 | .629 |
| GOF: $\chi^{2}$(4) = 4.234, p = .375. | | |

**Table S2**

Descriptive statistics for actor attributes in village 1, including only men present in at least two time waves (*n* = 60 at *T*_1_ , 74 at *T*_2_, 70 at *T*_3_).

| Variable | Mean (SD) | Range (Min-Max) | Density |
| --- | --- | --- | --- |
| \| Status *T*_1_ \| 5.45 (2.61) \| 9 (1– 10) \| - \| \| --- \| --- \| --- \| --- \| \| Status *T*_2_ \| 5.54 (2.77) \| 9 (1– 10) \| - \| \| Status *T*_3_ \| 6.05 (2.62) \| 9 (1– 10) \| - \| \| Strength & size *T*_1_^a^ \| 0.08 (0.77) \| 3.64 (-1.55– 2.10) \| - \| \| Strength & size *T*_2_^a^ \| 0.03 (0.78) \| 3.62 (-1.29 – 2.33) \| - \| \| Income *T*_1_ \| 5.43 (2.69) \| 9 (1– 10) \| - \| \| Income *T*_2_ \| 5.65 (2.75) \| 9 (1– 10) \| - \| \| Log age *T*_1_ \| 1.484 (0.17) \| 0.66 (1.20– 1.86) \| - \| \| Log age *T*_2_ \| 1.56 (0.14) \| 0.57 (1.32– 1.89) \| - \| \| Close kin *T*_1_ \| 8.87 ^b^ \| 24 (0– 24) ^c^ \| 0.06^d^ \| \| Close kin *T*_2_ \| 10.60 ^b^ \| 22 (0– 22) ^c^ \| 0.08^d^ \| \| ^a^ Standardized values.  ^b^ Mean degree.  ^c^ Range of degree (min degree – max degree).  ^d^Density of ties between close kin. \| \| \| \| | | | |

**Table S3**

Network and Covariate effects included in the objective functions of the SAOM model.

|  | | | |
| --- | --- | --- | --- |
| **Effect** | **Mathematical Expression** | **Graphical Expression** | **Interpretation** |
| **Structural Network Effects** |  |  |  |
| Out-degree (density) | $\sum_{j} x_{ij}$ | 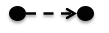 | The average tendency for actors to nominate others as cooperating with them. |
| Reciprocity | $\sum_{j} x_{ij}x_{ji}$ | 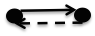 | The average tendency for actors to reciprocate cooperation nominations$.$ |
| Transitive group formation (gwespFF) | ${\sum_{k=1}^{n-2} x}_{ij}e^{\alpha}\{1-\left( 1-e^{-\alpha} \right)\sum_{h=1}^{n} x_{ih}x_{hj}\}$ | 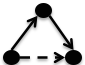 | The average tendency for transitive closure in the cooperation network. |
| In-degree Popularity (Sqrt) | $\sum_{j} x_{ij}\sqrt{x_{+j}}$ | 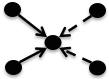 | The average tendency for actors to attract greater incoming ties from alters due to their high current in-degree ties. |
| Out-degree Activity (Sqrt) | $x_{i+}\sqrt{x_{i+}}$ | 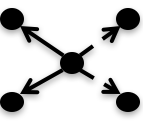 | The average tendency for actors to create out-degree ties due to their high current out-degree ties. |
| **Covariate Effects** |  |  |  |
| Main effect (centered) of the dyadic covariate | $\sum_{j} x_{ij}\left( w_{ij}-\bar{w} \right)$ | 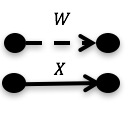 | The effect of having a kinship tie with an actor in the kinship network on forming a tie with that actor in the cooperation network. |
| Covariate outdegree (ego) | $\sum_{j} x_{ij}v_{i}$ | 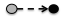 | The tendency for actors with high values on a covariate to create greater out-degree ties. |
| Covariate indegree (alter) | $\sum_{j} x_{ij}v_{j}$ | 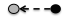 | The tendency for actors with high values on a covariate to receive greater in-degree ties. |
| Covariate Similarity | $\sum_{j} x_{ij}\left( {sim}_{ij}-\bar{sim} \right)$ | 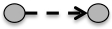 | The tendency for actors to create ties with alters who have similar values to them on a given covariate. |
| **Effects on status dynamics** |  |  |  |
| Linear shape | $z_{i}$ |  | Expresses the basic drive towards high values on status. |
| Quadratic shape | $z_{i}^{2}$ |  | Represents the feedback effect of status on itself. |
| Average alter | $z_{i}\left( \sum_{j} x_{ij}z_{j} \right)$/$\left( \sum_{j} x_{ij} \right)$ |  | The tendency for actors to increase their status due to the influence of the average of their alters’ statuses. |
| Main covariate effect | $z_{i}v_{i}$ |  | The main effect of an actor’s score on a given covariate on their status (status is centered and has an overall mean of 0). |

| **Table S4**  Model including estimated effects of network structure and covariates on Cooperation Network Dynamics and Status Dynamics in village 1, from a network-behaviour coevolutionary SAOM (stochastic actor-oriented model). | | | | |
| --- | --- | --- | --- | --- |
| Parameter | *β* | *SE* | *p* | *OR [CI]* |
| Cooperation Network Dynamics |  |  |  |  |
| Cooperation rate (period 1) | 34.48 | 8.20 | <.001 | - |
| Cooperation rate (period 2) | 12.34 | 1.43 | <.001 | - |
| Outdegree (density) | 0.42 | 0.51 | 0.414 | 1.52 [0.56, 4.16] |
| Reciprocity | 1.48 | 0.13 | <.001 | 4.39 [3.42, 5.64] |
| Tendency towards transitivity ^a^ | 1.37 | 0.10 | <.001 | 3.93 [3.24, 4.76] |
| Indegree popularity (sqrt) | -0.54 | 0.12 | <.001 | 0.58 [0.46, 0.73] |
| Outdegree activity (sqrt) | -0.50 | 0.13 | <.001 | 0.61 [0.47, 0.78] |
| Main effect of close kinship | 0.62 | 0.09 | <.001 | 1.86 [1.54, 2.24] |
| Status indegree | 0.06 | 0.03 | 0.046 | 1.06 [1.00, 1.13] |
| Status outdegree | 0.11 | 0.05 | 0.025 | 1.11 [1.01, 1.23] |
| Status similarity | 0.33 | 0.22 | 0.132 | 1.40 [0.90, 2.16] |
| Strength and size indegree | 0.17 | 0.06 | 0.006 | 1.19 [1.05, 1.35] |
| Strength and size outdegree | -0.03 | 0.07 | 0.664 | 0.97 [0.84, 1.12] |
| Strength and size similarity | 0.29 | 0.23 | 0.204 | 1.34 [0.85, 2.11] |
| Income indegree | -0.02 | 0.02 | 0.296 | 0.98 [0.95, 1.02] |
| Income outdegree | 0.00 | 0.02 | 0.866 | 1.00 [0.95, 1.04] |
| Income similarity | 0.24 | 0.14 | 0.080 | 1.27 [0.97, 1.67] |
| Log age indegree | -0.13 | 0.33 | 0.69 | 0.88 [0.46, 1.67] |
| Log age outdegree | -3.45 | 0.72 | <.001 | 0.03 [0.01, 0.13] |
| Log age similarity | -0.54 | 0.22 | 0.015 | 0.58 [0.38, 0.90] |
|  |  |  |  |  |
| Status Dynamics |  |  |  |  |
| Status rate (period 1) | 7.00 | 1.63 | <.001 | - |
| Status rate (period 2) | 5.25 | 1.37 | <.001 | - |
| Status linear shape | -0.16 | 0.12 | 0.162 | 0.85 [0.67, 1.07] |
| Status quadratic shape | -0.06 | 0.03 | 0.037 | 0.94 [0.89, 1.00] |
| Status total alter | 0.07 | 0.03 | 0.017 | 1.07 [1.01, 1.13] |
| Strength and size | 0.19 | 0.10 | 0.047 | 1.21 [1.00, 1.47] |
| Income | 0.07 | 0.03 | 0.011 | 1.07 [1.02, 1.13] |
| Log age | 1.52 | 0.66 | 0.022 | 4.55 [1.25, 16.59] |
| ^a^ Tendency towards transitivity was measured using the geometrically weighted shared edgewise partners effect (GWESP), which has an *α* = .69.  All *p* values are two-tailed. | | | | |

| **Table S5**  Model including estimated effects of network structure and covariates on Cooperation Network Dynamics and Status Dynamics in village 1, from a network-behaviour coevolutionary SAOM (stochastic actor-oriented model). | | | | |
| --- | --- | --- | --- | --- |
| Parameter | *β* | *SE* | *p* | *OR [CI]* |
| Cooperation Network Dynamics Cooperation rate (period 1) | 33.81 | 5.79 | <.001 | - |
| Cooperation rate (period 2) | 12.32 | 1.50 | <.001 | - |
| Outdegree | 0.36 | 0.48 | 0.454 | 1.43 [0.56, 3.7] |
| Reciprocity | 1.48 | 0.13 | <.001 | 4.40 [3.40, 5.68] |
| Tendency towards transitivity ^a^ | 1.37 | 0.10 | <.001 | 3.91 [3.22, 4.76] |
| Indegree popularity (sqrt) | -0.54 | 0.11 | <.001 | 0.58 [0.47, 0.73] |
| Outdegree activity (sqrt) | -0.48 | 0.12 | <.001 | 0.62 [0.49, 0.79] |
| Main effect of close kinship | 0.62 | 0.09 | <.001 | 1.85 [1.54, 2.22] |
| Status indegree | 0.06 | 0.03 | 0.046 | 1.06 [1.00, 1.13] |
| Status outdegree | 0.11 | 0.05 | 0.025 | 1.11 [1.01, 1.22] |
| Status similarity | 0.32 | 0.22 | 0.158 | 1.37 [0.88, 2.13] |
| Strength and size indegree | 0.18 | 0.06 | 0.004 | 1.19 [1.06, 1.34] |
| Strength and size outdegree | -0.03 | 0.08 | 0.675 | 0.97 [0.83, 1.13] |
| Strength and size similarity | 0.30 | 0.22 | 0.176 | 1.35 [0.87, 2.10] |
| Income indegree | -0.02 | 0.02 | 0.318 | 0.98 [0.95, 1.02] |
| Income outdegree | -0.01 | 0.02 | 0.783 | 0.99 [0.95, 1.04] |
| Income similarity | 0.25 | 0.13 | 0.061 | 1.29 [0.99, 1.67] |
| Log age indegree | -0.13 | 0.33 | 0.696 | 0.88 [0.46, 1.67] |
| Log age outdegree | -3.40 | 0.70 | <.001 | 0.03 [0.01, 0.13] |
| Log age similarity | -0.51 | 0.22 | 0.02 | 0.60 [0.39, 0.92] |
|  |  |  |  |  |
| Status Dynamics |  |  |  |  |
| Status rate (period 1) | 7.01 | 2.07 | 0.001 | - |
| Status rate (period 2) | 5.44 | 1.47 | <.001 | - |
| Status linear shape | -0.08 | 0.09 | 0.408 | 0.93 [0.78, 1.11] |
| Status quadratic shape | -0.06 | 0.03 | 0.054 | 0.94 [0.89, 1.00] |
| Status average in-alter | 0.07 | 0.03 | 0.019 | 1.07 [1.01, 1.14] |
| Strength and size | 0.16 | 0.10 | 0.089 | 1.17 [0.98, 1.41] |
| Income | 0.07 | 0.03 | 0.009 | 1.07 [1.02, 1.12] |
| Log age | 1.13 | 0.54 | 0.037 | 3.10 [1.07, 8.99] |
| ^a^ Tendency towards transitivity was measured using the geometrically weighted shared edgewise partners effect (GWESP), which has an *α* = .69.  All *p* values are two-tailed. | | | | |

| **Table S6**  Model including estimated effects of network structure and covariates on Cooperation Network Dynamics and Status Dynamics in village 1, from a network-behaviour coevolutionary SAOM (stochastic actor-oriented model). | | | | |
| --- | --- | --- | --- | --- |
| Parameter | *β* | *SE* | *p* | *OR [CI]* |
| Cooperation Network Dynamics |  |  |  |  |
| Cooperation rate (period 1) | 33.67 | 5.56 | <.001 | - |
| Cooperation rate (period 2) | 12.28 | 1.33 | <.001 | - |
| Outdegree | 0.24 | 0.41 | 0.563 | 1.27 [0.57, 2.8] |
| Reciprocity | 1.48 | 0.12 | <.001 | 4.40 [3.47, 5.58] |
| Tendency towards transitivity ^a^ | 1.38 | 0.10 | <.001 | 3.96 [3.28, 4.79] |
| Indegree popularity (sqrt) | -0.52 | 0.11 | <.001 | 0.59 [0.48, 0.74] |
| Outdegree activity (sqrt) | -0.46 | 0.11 | <.001 | 0.63 [0.52, 0.78] |
| Main effect of close kinship | 0.61 | 0.09 | <.001 | 1.84 [1.53, 2.21] |
| Status indegree | 0.06 | 0.03 | 0.049 | 1.06 [1.00, 1.13] |
| Status outdegree | 0.10 | 0.04 | 0.019 | 1.11 [1.02, 1.21] |
| Status similarity | 0.32 | 0.22 | 0.145 | 1.38 [0.89, 2.14] |
| Strength and size indegree | 0.17 | 0.06 | 0.004 | 1.19 [1.06, 1.34] |
| Strength and size outdegree | -0.03 | 0.07 | 0.681 | 0.97 [0.84, 1.12] |
| Strength and size similarity | 0.30 | 0.23 | 0.196 | 1.35 [0.86, 2.11] |
| Income indegree | -0.02 | 0.02 | 0.381 | 0.98 [0.95, 1.02] |
| Income outdegree | -0.01 | 0.02 | 0.788 | 0.99 [0.95, 1.04] |
| Income similarity | 0.24 | 0.14 | 0.078 | 1.27 [0.97, 1.67] |
| Log age indegree | -0.12 | 0.33 | 0.713 | 0.89 [0.46, 1.70] |
| Log age outdegree | -3.33 | 0.68 | <.001 | 0.04 [0.01, 0.14] |
| Log age similarity | -0.53 | 0.23 | 0.02 | 0.59 [0.38, 0.92] |
|  |  |  |  |  |
| Status Dynamics |  |  |  |  |
| Status rate (period 1) | 7.72 | 2.36 | 0.001 | - |
| Status rate (period 2) | 6.63 | 1.76 | <.001 | - |
| Status linear shape | 0.03 | 0.06 | 0.651 | 1.03 [0.91, 1.16] |
| Status quadratic shape | -0.03 | 0.02 | 0.096 | 0.97 [0.94, 1.01] |
| Status total in-alter | 0.16 | 0.08 | 0.045 | 1.18 [1.00, 1.38] |
| Strength and size | 0.18 | 0.08 | 0.022 | 1.19 [1.03, 1.39] |
| Income | 0.06 | 0.02 | 0.006 | 1.06 [1.02, 1.11] |
| Log age | 0.84 | 0.44 | 0.055 | 2.30 [0.98, 5.40] |
| ^a^ Tendency towards transitivity was measured using the geometrically weighted shared edgewise partners effect (GWESP), which has an *α* = .69.  All *p* values are two-tailed. | | | | |

**Table S7**

Descriptive statistics for actor attributes in village 2 (*n*=89).

| Variable | Mean (SD) | Range (Min-Max) | Density |
| --- | --- | --- | --- |
| Status | 5.48 (2.86) | 9 (1– 10) |  |
| Strength & size^a^ | 0.00 (0.72) | 3.81 (-1.75– 2.06) | - |
| Income | 5.51 (2.85) | 9 (1– 10) | - |
| Log age | 1.55 (0.14) | 0.59 (1.32– 1.91) | - |
| Close kin | 12.9^b^ | 34 (0– 34)^c^ | 0.07^d^ |
| ^a^ Standardized values.  ^b^ Mean degree.  ^c^ Range of degree (min degree – max degree).  ^d^Density of ties between close kin. | | | |

| **Table S8**  Parameter estimates (θ), Standard error, *p*-values and odds ratios for the ERG model assessing village 2 (*n*=89). | | | | |
| --- | --- | --- | --- | --- |
| Parameter | *θ* | *SE* | *p* | *OR [CI]* |
| Edges (outdegree) | -4.09 | 0.58 | <.001 | 0.02 [0.01, 0.05] |
| Mutual (Reciprocity) | 1.41 | 0.19 | <.001 | 4.09 [2.80, 5.97] |
| Tendency towards transitivity^a.^ | 1.04 | 0.11 | <.001 | 2.82 [2.28, 3.49] |
| Main effect of close kinship | 1.76 | 0.09 | <.001 | 5.83 [4.86, 7.00] |
| Status indegree | 0.04 | 0.02 | 0.025 | 1.05 [1.01, 1.09] |
| Status outdegree | -0.05 | 0.02 | 0.007 | 0.95 [0.92, 0.99] |
| Strength and size indegree | 0.25 | 0.07 | <.001 | 1.28 [1.12, 1.47] |
| Strength and size outdegree | -0.13 | 0.07 | 0.073 | 0.88 [0.77, 1.01] |
| Income indegree | 0.01 | 0.02 | 0.779 | 1.01 [0.97, 1.04] |
| Income outdegree | 0.02 | 0.02 | 0.193 | 1.02 [0.99, 1.06] |
| Log age indegree | -0.53 | 0.41 | 0.194 | 0.59 [0.27, 1.31] |
| Log age outdegree | 0.15 | 0.37 | 0.681 | 1.16 [0.56, 2.41] |
| Null deviance = 10857, *df* = 7832  Residual deviance = 2681, *df* = 7819  AIC = 2707  BIC = 2797 | | | | |
| ^a.^ GWESP (Geometrically weighted edgewise shared partners, decay = 0.1).  *CI* = 95% confidence intervals | | | | |

| **Table S9**  Parameter estimates (θ), Standard error, *p*-values and odds ratios for the ERG model assessing the subset of village 2 with complete data (*n*=67). | | | | | | |
| --- | --- | --- | --- | --- | --- | --- |
| Parameter | *θ* | *SE* | | | *p* | *OR [CI]* |
| Edges (outdegree) | -5.02 | 0.06 | | | 0 | 0.01 [0.01, 0.01] |
| Mutual (Reciprocity) | 1.22 | 0.24 | | | 0 | 3.39 [2.11, 5.45] |
| Tendency towards transitivity^a.^ | 1.03 | 0.14 | | | 0 | 2.79 [2.14, 3.63] |
| Main effect of close kinship | 1.80 | 0.12 | | | 0 | 6.06 [4.81, 7.63] |
| Status indegree | 0.01 | 0.01 | | | 0.016 | 1.01 [1.00, 1.03] |
| Status outdegree | -0.02 | 0.01 | | | 0.003 | 0.98 [0.97, 0.99] |
| Strength and size indegree | 0.03 | 0.01 | | | 0.002 | 1.03 [1.01, 1.05 |
| Strength and size outdegree | -0.03 | 0.01 | | | 0.004 | 0.97 [0.96, 0.99] |
| Income indegree | 0.01 | 0.02 | | | 0.73 | 1.01 [0.96, 1.06] |
| Income outdegree | 0.08 | 0.03 | | | 0.002 | 1.08 [1.03, 1.13] |
| Log age indegree | -0.52 | 0.58 | | | 0.372 | 0.60 [0.19, 1.85] |
| Log age outdegree | 0.66 | 0.54 | | | 0.228 | 1.92 [0.66, 5.59] |
| Null deviance = 6130, *df* = 4422  Residual deviance = 1692, *df* = 4409  AIC = 1718  BIC = 1801 | | |  |  |  |  |
| ^a.^ GWESP (Geometrically weighted edgewise shared partners, decay = 0.1).  *CI* = 95% confidence intervals | | | | | | |

**SM References**

1. Ripley R, Snijders T, Boda Z, Voros A, Preciada P. 2018 Manual for RSiena. <https://www.stats.ox.ac.uk/~snijders/siena/RSiena_Manual.pdf>.
2. Snijders T. 2001 The statistical evaluation of social network dynamics. *Sociol. Methodol.* **31**, 361-395.
3. Snijders T. 2009 Longitudinal methods of network analysis. In *Encyclopedia of Complexity and System Science* (ed R Meyers), pp. 5998-6013. New York: Springer.
4. Snijders T. 2016 The multiple flavours of multilevel issues for networks. In *Multilevel network analysis for the social sciences* (eds E Lazega, T Snijders), pp. 15-46. New York: Springer.
5. Snijders T, Steglich C, Schweinberger M. 2017 Modeling the coevolution of networks and behavior. In *Longitudinal Models in the Behavioral and Related Sciences* (eds K van Montfort, J Oud, A Satorra), pp. 41-71. Mahwah, NJ: Lawrence Erlbaum.
6. Block P, Koskinen J, Hollway J, Steglich C, Stadtfeld C. 2018 Change we can believe in: comparing longitudinal network models on consistency, interpretability and predictive power. *Soc. Networks* **52**, 180-191.
7. Snijders T, Van de Bunt G, Steglich C. 2010 Introduction to stochastic actor-based models for network dynamics. *Soc. Networks* **32**, 44-60.
8. Robins G, Pattison P, Kalish Y, Lusher D. 2007 An introduction to exponential random graph (p*) models for social networks. *Soc. Networks* **29**, 173-191.
9. Lusher D, Koskinen J, Robins G. 2013 *Exponential random graph models for social networks: Theory, methods, and applications*. Cambridge, UK: Cambridge University Press.
10. Snijders T, Pattison P, Robins G, Handcock M. 2006. New specifications for exponential random graph models. *Sociol. Methodol.* **36**, 99-153.
11. Hoff P. 2018 "*Package ‘sbgcop’*".
12. Hollenbach F, et al. 2014 Principled imputation made simple: multiple imputation using Gaussian copulas, *arXiv preprint arXiv:1411.0647*.
13. Handcock M, Hunter D, Butts C, Goodreau S, Morris M. 2003 “*statnet: Software tools for the Statistical Modeling of Network Data”*.
14. R Core Team. 2017 *R: A language and environment for statistical computing*. R Foundation for Statistical Computing, Vienna, Austria.
15. Goodreau S, Handcock M, Hunter D, Butts C, Morris M. 2008 A statnet Tutorial. *J. Stat. Softw.* **24**, 1-27.
16. Morris M, Handcock M, Hunter D. 2008 Specification of exponential-family random graph models: terms and computational aspects. *J. Stat. Softw.* **24**, 1548-7660.
